# Vaccination coverage in children can be estimated from health insurance data

**DOI:** 10.1186/1471-2458-8-82

**Published:** 2008-03-01

**Authors:** Helen Kalies, Rebekka Redel, Rudolf Varga, Martin Tauscher, Rüdiger von Kries

**Affiliations:** 1 Institute for Social Paediatrics and Adolescent Medicine, Munich, Germany; 2 Kassenärztliche Vereinigung Bayerns, Munich, Germany; 3 AOK Bayern – Die Gesundheitskasse, Munich, Germany

## Abstract

**Background:**

The introduction of new vaccines for young children requires instruments for a rapid and timely assessment of the progressively increasing vaccination coverage. We assessed whether routine data generated by statutory health insurances (SHI) might be used to monitor vaccination coverage in young children.

**Methods:**

For 90% of the population Germany's healthcare system is premium-funded through SHI. Specific medical codes on childhood vaccination are used for billing. These were used to analyse vaccine uptake up to 24 months in children born in Bavaria between 2001–10–01 and 2002–12–31. For children insured in the biggest SHI, vaccination coverage estimates based on billing data were compared to estimates considering only continuously insured children since birth, based on additional data provided by this SHI.

**Results:**

Definition of an appropriate denominator from the billing data was a major challenge: defining the denominator by any consultation by children with different ID numbers yielded 196,732 children, exceeding the number of births in Bavaria by a factor of 1.4. The main causes for this inflated denominator were migration and change of health insurance number. A reduced dataset based on at least one physician's visit in the first six months and 2nd year of life yielded 111,977 children. Vaccination coverage estimates for children in the biggest SHI were at maximum 1.7% higher than in the data set based on continuously insured children.

**Conclusion:**

With appropriate adjustments to define the denominator physician's billing data provide a promising tool to estimate immunisation coverage. A slight overestimation based on these data was explained by children never seeing a physician.

## Background

The WHO European Region strategic plan aims not only to attain high levels of childhood immunisations but also to provide maximum protection at an earliest possible age and to reduce geographical disparities in vaccination coverage [1]. Despite the fact that Germany has acknowledged these goals, this country has one of the lowest vaccination coverage rates in Europe [2], the administration of all recommended vaccines in children is considerably delayed and a high geographical variance between its 16 states exists (e.g. range for 1^st ^dose measles immunisation at 2 years: 58 – 88%) [3].

Although the need to establish a system for monitoring completeness and timeliness of immunisations is acknowledged, in many countries there are no registries for such data [4,5]. Therefore estimation of vaccination coverage in most countries is based on either ad hoc surveys of representative samples or on assessment during school health examinations or both [6-8]. Despite the undoubted usefulness of these data in the absence of better sources, they have several limitations: surveys are fraught with problems regarding completeness, sample size, representativity and immense costs, and school health examinations lag 5 to 6 years behind the current vaccination practice and do not assess types of vaccines or timing of vaccinations.

In countries like Germany, France and the US, childhood immunisations are mainly given by the private sector (paediatricians and general practitioners) [4,9]. Although these physicians get paid for vaccinating, imbursement data related to vaccination have not been used to assess vaccination coverage in a systematic manner.

In a cohort of children born between 2001–10–01 and 2002–12–31 in Bavaria, the second most densely populated German state, we therefore assessed whether such imbursement data might be useful to depict the vaccine uptake up to the age of 24 months.

## Methods

### The SHI system and the Association of SHI Physicians in Germany

Germany's healthcare system is premium-funded through statutory health insurances (SHI) for 90% of the population. There are about 115,000 physicians providing outpatient services within SHI in Germany. SHI pay for services of outpatient physicians through regional associations of SHI physicians which handle all billing information on an individual basis. To bill patients, outpatient physicians are obliged to be members of a regional branch of the 'Kassenärztliche Vereinigung' (KV). For payment, physicians submit electronic invoices based on medical codes for each visit to the KV. Any physicians' contacts and preventive measures, such as vaccinations, can be identified on the basis of a specific medical code. Each of the 16 states in Germany has at least one regional KV. Data of the Bavarian KV (KVB) allow identifying the different vaccine antigens administered at vaccination visits since July 2001.

### Patients and setting

Data of the KVB were used. Bavaria is the largest (70,552 km^2^) and the second most populated state of Germany with 12 million inhabitants [10]. We analysed physicians' consultations and vaccination histories for children born between 2001–10–01 and 2002–12–31 with a follow-up until 2004–12–31, allowing for analysis of any billing for these children up to an age of at least 24 months.

### Nature and structure of data

The KVB supplied data of all patient consultations that were billed by outpatient physicians on a quarterly basis. Each patient has a unique identification code within his SHI. This may change in case the patient changes the SHI, which does occur because of competition between SHI. The child's vaccination history therefore is completely mapped by the billing data unless the child switches to another SHI or to a physician outside of Bavaria during the observation period. The following data were available for the statistical analysis of the KVB data set: an identification code, member of the major health insurance company of Bavaria (AOK Bavaria; yes/no), birth date of the child, date of consultancy and – if vaccinated – 4- to 5-digit codes identifying the vaccines administered, or consultancy for any other reason per quartile year.

### Data analysis

To calculate vaccination coverage, the number of vaccinated children (numerator) and the total number of children eligible for vaccination (denominator) has to be known. While numerator data are directly provided in the KVB dataset, an appropriate denominator has to be derived from KVB data. A child not consulting a Bavarian outpatient physician or not insured in SHI will not appear in the KVB data. A simple approach to assess the number of children eligible for vaccination within the SHI is to consider all children with any physician billing up to 2004–12–31 (original KVB dataset). This approach does not take the following censoring events into account: (a) children migrating from or to Bavaria; (b) children treated by a Bavarian physician during their holidays and therefore leaving or entering responsibility of the KVB; (c) children changing between different SHI or between private health insurances and SHI. The magnitude of these problems and its influence on vaccination coverage estimates was not known. We therefore compared two datasets: (1) the original KVB dataset, consisting of all children with at least one physicians' visit during the defined follow-up period; and (2) a reduced KVB dataset, applying a simple algorithm which restricts the original dataset to a cohort of children with at least two physicians' visits, independent of its medical character.

### Validation

The number of children in the two KVB datasets for a Bavarian birth cohort born 2001–10–01 to 2002–12–31 is compared to the official numbers provided by the Bavarian Bureau of Statistics [10] and differences will be analysed.

Vaccination coverage is assessed both for the original and the reduced KVB dataset. Furthermore, to check if the reduction strategy of the KVB dataset was valid, additional information from one health insurance company of Bavaria (AOK) was obtained. The aim was to define a population that was continuously insured with one SHI during the observation period. The AOK Bavaria supplied data on all children born between 2001–10–01 and 2002–12–31 who were continuously insured in the AOK Bavaria from birth to 2004–12–31. The following data were available: an identification code, birth date of the child, date of entrance to and exit from the insurance. The vaccination histories of the children in the AOK dataset could be obtained by linking the AOK identification number to the identification number for AOK insured children in the KVB dataset. Vaccination coverage estimates from children continuously insured in the AOK Bavaria is compared to those from a reduced KVB dataset for AOK insured children only.

### Definitions for vaccination status

According to the German vaccination schedule [11] we defined a child as 'fully primed' if he or she received at least three doses of a vaccine containing acellular pertussis components or two doses of a vaccine not containing acellular pertussis components. A child was defined as 'fully immunized' if she or he received a booster dose at the age of 11 months or later following full priming. In accordance to the above definitions, we estimated the coverage of the following combined series: first dose, full priming and full immunisation against diphtheria, tetanus, pertussis, polio, *Haemophilus influenzae *type b and hepatitis B; first dose against measles, mumps and rubella (MMR) [7]. Uptake and timing of immunisation by age in months was calculated according to the Kaplan-Meier method which is described in detail by Laubereau et al. [12].

All data provided by the KVB and analysed in the Institute for Social Pediatrics and Adolescent Medicine, Munich, were completely anonymous in accordance with German data protection laws.

## Results

The original KVB dataset yields data on 196,732 children born between 2001–10–01 and 2002–12–31. This is more than the respective number of children born in Bavaria in the observation period (n = 142,809) [10]. The inflated denominator can be explained by (1) children migrating to Bavaria or consulting a Bavarian physician in their holidays and (2) children changing health insurance companies; these children will be recorded more than once because of changing insurance numbers. The timing of these censoring events is not recorded in the KVB datasets and therefore cannot be considered in the analysis. In order to deal with this problem, we reduced the original KVB dataset. Since we were interested in vaccine uptake in the first two years of life we required one visit in the first half of life and one in the second year of life – in order to make sure that this identified cohort was followed-up until the second year of life. This algorithm yielded a dataset with 111,799 children, corresponding to 78% of all children born in Bavaria during the observation period.

The reduced KVB dataset cohort is smaller than the respective birth cohorts defined by official data [10]. The difference in the number of children in the reduced dataset compared to the respective Bavarian birth cohort and the magnitude of different causes is shown in table 1: (1) children who are privately insured can not appear in the KVB dataset, (2) children who change insurance and therefore could not be followed-up will not appear in the reduced dataset, (3) children who never consulted an outpatient physician or not consulted an outpatient physician in their first six months and in the second year of life will not appear in the reduced dataset, nor will (4) children who move from Bavaria.

**Table 1:** The reduced KVB dataset compared to the respective Bavarian birth cohorts and causes for the observed differences.

	percent	N
Bavarian birth cohorts (1.10.2001–31.12.2002) [9]	100	142,809
Reduced KVB dataset	78	111,977

difference		30,832

Reasons:		
privately insured^+^	10.0	14,281
change of insurance*	5.0–8.0	7,140–11,425
no consultancy of outpatient physician in 1^st ^6 months and 2^nd ^year of life*	4.9	6,998
moving from Bavaria [9]	1.4	1,999

* estimated from AOK Bavaria data (own data)

^+ ^personal communication of Dr. Frank Niehaus, WIP-PKV (scientific Institute of the Private Health Insurances)

The number of children in the validation AOK dataset was 40,645. 95.1% of the children in the validation dataset had visited an outpatient physician in the first six months of life and in the second year of life, which was the algorithm to generate the reduced KVB dataset from the original KVB dataset. The proportion of children not consulting a physician in both periods was 3.1%, the proportion of children never visiting a physician was 1.8%.

The reduced KVB dataset on AOK insured children (n = 44,755), however, was even greater than the number of children in the provided AOK dataset; this is explained by children in the reduced KVB dataset with at least one physician's visit in the second year of life who may have left the AOK before 2004–12–31 whereas the validation dataset required all children to be continuously insured from birth to 2004–12–31.

In table 2 point estimates of vaccination coverage with 95% confidence intervals derived from the four different datasets are shown: the original and the reduced KVB dataset; a subset of the reduced KVB dataset which only includes AOK insured children; and a dataset for children continuously insured in the AOK (validation dataset). The vaccination coverage estimates of the reduced KVB dataset were lower than those for the original KVB dataset. The reduced KVB dataset on AOK insured children and the AOK validation dataset point to slightly higher vaccination coverage estimates in AOK insured children. In the reduced KVB dataset on AOK Bavaria insured children the vaccination coverage estimates were up to 1.7% higher than those for the AOK validation dataset with disjunctive 95% confidence intervals.

**Table 2:** Comparison of vaccination coverage estimates (%; 95% CI) between the two KVB datasets, the dataset for AOK insured children in the reduced KVB cohort and the validation data set for two different vaccinations at different children's ages.

		Vaccination coverage (%)			
		
			DTPPolioHibHep		
			
Data Set	N	MMR 1^st ^dose at 2 y	1^st ^dose at 3 months	Full primed at 1 y	Full immunized at 2 y
Original KVB dataset	196,732	60.4	14.6	45.7	40.4
		60.2–60.7	14.4–14.8	45.5–45.9	40.2–40.7
Reduced KVB dataset	111,977	75.4	22.3	67.5	56.0
		75.1–75.6	22.1–22.6	67.0–67.6	55.7–56.3
Subset of the reduced KVB data set (AOK Bavaria insured only)	44,755	77.2	24.1	70.5	57.7
		76.8–77.6	23.7–24.5	70.1–71.0	57.3–58.2
Validation data set	40,645	76.0	23.1	68.8	56.6
		75.5–76.4	22.7–23.5	68.3–69.2	56.1–57.1

**Figure 1:**
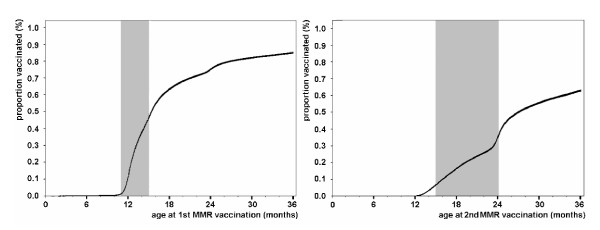
Vaccination covarage estimates for MMR derived from the reduced KVB dataset. Inverse Kaplan-Meier curves (95% CI) indicating the proportion of children vaccinated by age of child at first (left) or second (right) MMR vaccination. Shaded areas mark the recommended age for the respective dose.

Figure 1 shows uptake and timing of the first and second MMR immunisation by age using inverse Kaplan-Meier curves is shown in relation to the age recommended by the German Standing Committee on Vaccination [11].

## Discussion

These data from Bavaria show that using routine data generated by SHI to handle physicians' billings may provide a useful tool to estimate immunisation uptake in the first two years of life in Germany.

The main challenge in using routine data generated by SHI to handle physicians' billings is to define an appropriate denominator. Considering all different insurance numbers related to any physicians' billing as a surrogate for the number of children eligible for vaccination (original KVB dataset) will yield to a huge overestimation of the denominator and therefore to an underestimation of vaccination coverage estimates.

A restriction to children with at least two visits – one in the first six months and one in the second year of life – reduces the original dataset to 78% of the size of the Bavarian birth cohort. The impact of this algorithm could be assessed with the AOK validation dataset: only 3.1% of all children did not visit an outpatient physician in these two periods. The high proportion of children seen by a physician in their first two years of life might be a consequence of free of charge well baby check-up visits in the first two years (U1-U7) with a compliance of 80% – 90% for each check-up [13].

The vaccination coverage estimates for the reduced KVB dataset on AOK insured children gave similar vaccination coverage estimates as for the AOK validation dataset. These were, however, up to 1.7% higher than for the AOK validation dataset. This systematic overestimation of vaccination coverage based on KVB data might be explained by children never consulting an outpatient physician – some 1.8% of the children in the AOK validation dataset. These children will not be vaccinated and – as they are not included in the denominator of the reduced KVB dataset – vaccination coverage is slightly overestimated in the reduced KVB dataset. It might be argued to confine vaccination coverage estimates to the app. 30% AOK insured children in Bavaria. Unfortunately such data are not routinely available – they were generated on special request for this study.

The vaccination coverage estimates presented here are not based on the entire Bavarian birth cohort. In table 1 causes for the missing 22% were plausibly explained. There are no data, however, to suggest that vaccination coverage for children in families moving out of Bavaria and those changing SHI should be different from the entire cohort. Vaccination coverage of privately insured children might differ since these children are from families who are exempted from the SHI system because their parents' income exceeds a certain threshold. The direction of the possible bias for vaccination coverage in this subgroup, however, is difficult to estimate: while parents with predominantly higher education levels might be more careful to have there children vaccinated in time, this clientele also includes parents attracted by 'alternative' medicine who are reluctant about vaccinations.

The vaccination coverage estimates generated from billing data for SHI insured children allow depicting the vaccine uptake as a time dependent variable. As shown exemplarily in the figure, the MMR vaccination has not only a low coverage with 24 months of age, but also exists a considerably delay in its administration: at the end of the recommended age (15 and 24 months for the first and second dose, respectively) less than 50% of the children were vaccinated with the respective dose. A similar picture appears for all other generally recommended vaccines (data not shown). Comparably low and delayed vaccination coverage estimates in young children have been shown by a national immunisation survey [3], but when restricting these data to Bavarian children only extremely wide confidence intervals derive.

## Conclusion

The outlined use of SHI billing data to estimate vaccination coverage appears to be a promising approach for low cost in countries where vaccinations are administered by the private sector. The size of a systematic overestimation of vaccination coverage due to children never seeing an outpatient physician and therefore not appearing in datasets based on billing data was found to be small. This data source might provide a useful tool for rapid assessment of progressively increasing vaccination uptake by newly introduced vaccines.

## Competing interests

The author(s) declare that they have no competing interests.

## Authors' contributions

HK coordinated and designed the study, analysed and interpreted the data, drafted the manuscript

RR analysed part of the data and revised the manuscript critically for important intellectual content

RV revised the manuscript critically for important intellectual content

MT analysed part of the data and revised the manuscript critically for important intellectual content

RvK made substantial contributions to conception and design of the study and revised the manuscript critically for important intellectual content

All authors read and approved the final manuscript

## Pre-publication history

The pre-publication history for this paper can be accessed here:


